# A Comprehensive Review of Characterization Methods for Metallurgical Coke Structures

**DOI:** 10.3390/ma15010174

**Published:** 2021-12-27

**Authors:** Heng Zheng, Runsheng Xu, Jianliang Zhang, Oday Daghagheleh, Johannes Schenk, Chuanhui Li, Wei Wang

**Affiliations:** 1 The State Key Laboratory of Advanced Metallurgy, University of Science and Technology Beijing, Beijing 100083, China; heng.zheng@stud.unileoben.ac.at (H.Z.); jl.zhang@ustb.edu.cn (J.Z.); jgzmhui@foxmail.com (C.L.); 2 Chair of Ferrous Metallurgy, Montanuniversitaet Leoben, Franz-Josef-Straβe 18, 8700 Leoben, Austria; daghagheleh@unileoben.ac.at (O.D.); johannes.schenk@unileoben.ac.at (J.S.); 3 The State Key Laboratory of Refractories and Metallurgy, Wuhan University of Science and Technology, Wuhan 430081, China; wangwei74@wust.edu.cn

**Keywords:** coke quality, coke structures, characterization, molecular model

## Abstract

The structure of coke affects its reactivity and strength, which directly influences its performance in the blast furnace. This review divides coke structures into chemical structure, physical structure, and optical texture according to their relevant characteristics. The focuses of this review are the current characterization methods and research status of the coke structures. The chemical structures (element composition and functional group) can be characterized by elemental analysis, Fourier transform infrared spectroscopy (FTIR), Raman spectroscopy (Raman), X-ray photoelectron spectroscopy (XPS), and nuclear magnetic resonance imaging technology (13C NMR). The physical structures (pore structure and micro-crystallite structure) can be characterized by image method, X-ray CT imaging technique, mercury intrusion method, nitrogen gas adsorption method, X-ray diffraction method (XRD), and high-resolution transmission electron microscopy (HRTEM). The optical textures are usually divided and counted by a polarizing microscope. In the end, this review provides an idea of the construction of a coke molecular structural model, based on the above characterization. With the coke model, the evolution principles of the coke can be calculated and simulated. Hence, the coke performance can be predicted and optimized.

## 1. Introduction

The blast furnace (BF) is a high-efficiency shaft furnace. The high heat utilization rate and production efficiency make the BF route the dominant ironmaking process globally [1]. About 70% of the hot metal used in producing crude steel comes from the BF route [2,3,4]. Coke is an indispensable burden in the blast furnace, and its performance shows a substantial impact on the ironmaking process [5]. The coke plays the role of the skeleton, which ensures the gas and liquid penetration through the blast furnace. The Coke reactivity index (CRI) and the coke strength after reaction (CSR) are the most widely used indexes to indicate the coke degradation potential in a blast furnace. A coke with good quality shows a lower CRI and a higher CSR.

To cut CO_2_ emissions during BF ironmaking process, many methods have been developed for substituting part of the coke, such as the application of ferro coke [6,7] and biomass coke [8,9], injection of pulverized coal or biomass, or natural gas, etc. [10,11,12,13,14]. Oxygen-enriched blast technology is another well-used method to decrease the coke ratio by increasing the production rate of hot metal through enhancing the combustion intensity of coke and pulverized coal [15,16]. However, these methods result in higher requirements of coke quality, especially the coke strength. The coke quality should be adequate to ensure a continuous production of hot metal in the BF [17].

Coke shows different deterioration degrees in different parts of the blast furnace. During the movement from the top to the lower part of the furnace, most lump cokes break into smaller particles consumed in the hearth. In this process, the structures of the coke change significantly [18]. It is well known that the reactivity [19,20] and the strength of coke [21,22,23,24] are closely related to its structures. Therefore, it is essential to characterize the coke structures comprehensively. It should be noted that the properties of the parent coal and coking condition also influence the coke properties. Currently, there is no specific test for coals to guarantee high-quality coke products. Instead, multitudes of empirical laboratory tests have been developed to investigate aspects of the physical, chemical, and thermoplastic behavior of individual coals and blends [25]. Some researchers summarized some models for predicting the metallurgical coke quality based on maceral composition and properties of coals. However, the models were not satisfactory when applied to coals or blends of different geological histories [26]. Generally, in terms of coal properties, coke quality is primarily influenced by coal rank, coal type (reactive and inert macerals), and its inherent ability to soften, become plastic, and re-solidify into a coherent mass when heated [25]. As a general comment on model replication, they cannot be used beyond the conditions under which they were derived [27]. It is more acceptable to conclude the relations between coke structure and the properties.

At present, many studies discussed the relationship between coke structures and properties, but there is a lack of systematic discussion on characterization methods of coke structures. Considering the complexity of coke structures, this review divides the coke structures into chemical structure, physical structure, and optical texture. First, the relation between coke structures and coke properties is reviewed. Then, the appropriate characterization methods of the coke structures are introduced. Lastly, a general procedure of construction of a coke molecular model is proposed. We hope to bring about some inspiration for understanding and simulating the coke degradation at a molecular scale.

## 2. Relations between Coke Structures and Properties

The most important properties of coke are the reactivity and the strength. To measure the reaction characteristics of coke under high temperature conditions, the reactivity index (CRI), and coke strength after reaction (CSR) are proposed. CRI refers to the ability of the coke to react with carbon dioxide at 1100 °C. CSR refers to the strength of the coke after the reaction with carbon dioxide. CRI and CSR are two critical indicators of the thermal performance of coke. It is believed that a good quality metallurgical coke should have low CRI and high CSR. If the CRI is too high, the starting temperature of the reaction between the coke and CO_2_ will decrease. In other words, the reaction may start at the upper part of the blast furnace, resulting in the generated CO escaping from the top of the blast furnace. The cold/hot compressive strength of the coke is measured by two flat plates in room/high temperature conditions. One of the plates moves to compress the coke sample till the coke is destroyed. When the coke is used in a giant blast furnace, the compressive strength should be sufficient to support the iron-containing burdens. The following subsections introduce the relations between coke structures and the coke properties.

### 2.1. Coke Structure and Reactivity

The main factors influencing the reactivity of coke include the pore structures, the mineral types and contents, and the inherent properties of the carbon matrix. Guo et al. [28] analyzed the porosities, pore wall thicknesses, and pore size distributions of 10 types of cokes to find the relations between these parameters and CRI. The reactivity of the coke was mainly affected by the content of pores with a size between 60 and 120 μm. These pores were important channels for the diffusion of the reaction gas into the coke. The coke with a larger pore wall thickness showed a lower CRI. Fott et al. [29] found a similar phenomenon in that the effective diffusion rate of CO_2_ in the ultra-micropores and macropores of the coke was relatively small. In these pores, the reaction only occurred in a small local area. 

There are two primary sources of minerals that affect coke’s reactivity: the inherent minerals in the coke matrix and the extrinsic minerals that are enriched in the blast furnace. A large number of studies have pointed out that the minerals in the coke matrix showed different promotion effects on the reactivity of the coke [30,31,32,33,34]. Generally, the cations of the minerals played a critical role in the promotion effects. The catalytic effect in descending order were K^+^ > Na^+^ > Ca^2+^ > Mg^2+^ > Fe^2+^ > Al^3+^ > Si^4+^. To study the impact of the specific types of the minerals, a so-called coke analogue was developed for simulating the industrial coke. Reid et al. [30] used the coke analogue to simulate the metallurgical coke with a particular type of mineral. It was found that K^+^ showed the most potent catalytic effect. Due to the strong catalytic effect, alkali elements are regarded as detrimental for the blast furnace [35,36]. The influence of alkali elements in the blast furnace on coke reactivity has been a hot topic [37]. The impact of Zn has also been extensively studied in recent years [38,39]. The extrinsic minerals enriched in the blast furnace are not within the scope of this review paper. In general, the appearance of these alkali and Zn elements could strongly accelerate the degradation of the coke. 

Coke is a brittle material with a developed pore system composed of pore walls, pores, and cracks. The basic unit of the coke pore wall is the microcrystallite structure [40]. Ignoring the influence of the pore structures and the mineral effect, the inherent property of the coke matrix is the decisive factor that determines the reactivity of the coke. The two crucial intrinsic properties of the coke matrix are microcrystallite and optical characteristics. The method of determining the microcrystallite parameters, such as the average stacking height (Lc) of crystalline, the average size (La) of crystalline layer, and the average spacing (d_002_) of crystalline, are introduced in Section 3.2.2. The method of determining optical characteristics, such as isotropic texture content (∑ISO) and anisotropic degree (OTI) is presented in Section 3.3. Zhang et al. [41] evaluated 30 different metallurgical cokes and found that d002, Lc, and La were in the ranges of 3.46–3.51 Å, 28.95–42.18 Å, and 41.40–53.05 Å, respectively. The interlayer spacing of cokes showed a neglectable difference. It was found that when the mineral effect was not strong, the CRI decreased with the increasing Lc and La. During the CO_2_ gasification reaction, the isotropic texture in the coke reacted more quickly than the anisotropic texture [42,43,44,45]. The higher value of the ∑ISO of the coke is, the higher CRI of the coke will be. OTI is another index but describes the same optical phenomenon of the coke matrix. Generally, the coke with a higher value of OTI shows lower CRI.

### 2.2. Coke Structure and Strength

The direct forces that cause the degradation of the coke in the blast furnace are the friction and collision between the bulk burden materials, such as iron ore sinter, iron ore pellet, and cokes. The coke strength can be comprehensively indicated by cold compressive strength, CSR, and hot compressive strength. The pore structures play an essential role in determining the strength of the coke. Mihashi et al. [46] found that the matrixes of different cokes showed similar strength. Young et al. [22] used the 3-dimensional discrete element method to simulate the compressive cracks of coke. It was found that porosity is the main factor affecting the strength of the coke. A uniform pore distribution and less porosity contributed to the coke’s higher cold compressive strength. Saito et al. [23] used the rigid bodies–spring model to analyze the relations between the pores and the cold compressive strength. He found that slight roundness and large distorted pores might cause stress concentration and significantly reduce the strength of coke. Gornostayev et al. [24] found a similar result by directly observing the coke using a scanning electron microscope. The elliptical, elongated, and flattened pores, compared with circular pores, showed a lower ability to resist load pressure. The high cold compressive strength of coke could not guarantee its high CSR. The CSR presents a good negative correlation with the CRI [21,47,48]. The factors that affect the CRI of the coke inevitably affect the CSR of coke. The aim of measuring hot compressive strength is to simulate the load borne by the coke in an actual blast furnace. Haapakangas et al. [49,50] used the Gleeble thermomechanical simulator to evaluate the coke hot compressive strength at 1000 °C, 1600 °C, and 1750 °C. It was found that the coke was brittle at 1000 °C but partially plastic at 1600 °C and 1750 °C. The high-temperature treatment above the temperature of the coking furnace may cause further graphitization. The non-graphitizing carbons became harder, and the graphitizing carbons became softer at temperatures between 1000 °C and 2000 °C. The low coke compressive strength at high temperature was considered to be due to the high graphitization degree of the coke. Fang et al. [51] investigated the relations between temperature, carbon loss of the coke, and hot compressive strength. It was found that the hot compressive strength was significantly decreased with the increasing carbon loss of the coke and the temperature. However, the effects of individual factors affecting coke hot strength were not illustrated. Guo et al. [52] studied the influence of the pore structure features on the hot compressive strength of coke. It showed that in the temperature between 1000 °C and 1300 °C, the temperature presented a limited influence on the strength. The coke with a higher hot compressive strength showed a smaller pore size with a more uniform distribution. The following chapter introduced the relevant characterization methods of the coke structures.

## 3. Characterization of Coke Structures

### 3.1. Chemical Structure

Coke is the solid residue of impure carbon obtained from coal after pyrolysis in a coking oven. The chemical structure of coke is discussed in two aspects: the element composition and the functional group. The chemical data obtained through experiments helps to obtain the formula of coke.

#### 3.1.1. Ultimate Analysis

Coke mainly consists of C, H, O, N, and S elements. The average molecular formula of a coke can be simply represented by the ratio of these five elements. The contents of C and H are determined by the combustion method. The principle is to convert C and H into CO_2_ and H_2_O. The generated CO_2_ and H_2_O can be captured by an absorbent. The C and H contents in the coke samples are calculated from the weight gain of the absorbent. The determination of N content is to add the mixed catalyst and sulfuric acid to the coke sample. The N will convert into NH_4_HSO_4_ after being heated. Then adding NaOH into the mixed liquid, the NH_3_ will be evaporated and absorbed into the H_3_BO_3_. The N content can be obtained by titration with the sulfuric acid standard solution. The determination of S content can be done by Eschka method, the bomb washing method, and the high-temperature combustion method. For O analysis, the methods are similar to those described for the determination of C but with pyrolysis in helium instead of oxygen. The easiest way to obtain O content is using 100% minus the total contents of C, H, N, and S. The contents of H, O, N, and S elements in the coke are typically low. White et al. [53] discussed how to determine these elements. Therefore, detailed procedures are not given here. It should be noted that there is also the proximate analysis of coke determining the content of fixed carbon, moisture, volatile matter, and ash of the coke. The proximate analysis provides a fast determination of the composition of the coke and gives a preliminary judgment of the coke quality. The ultimate analysis is more comprehensive and is dependent on quantitative analysis of C, H, O, N, and S elements present in the coke. The C content from the ultimate analysis is generally higher than the fixed carbon content from the proximate analysis. Because the C content from ultimate analysis includes the carbon that escapes as volatile matter. For the calculation of coke molecular formula, the ultimate analysis should be used.

The molecular formula of a coke can be calculated according to the ratio of H/C, S/C, O/C, and N/C obtained from the ultimate analysis. Wang et al. [54] and Tian et al. [55] have successfully determined the average molecular formula of coke based on the elemental ratio and built the molecular model. The detailed molecular model is discussed in Section 4.

#### 3.1.2. Functional Group

Most of the functional groups on the benzene rings decompose during high-temperature carbonization. At present, the following four methods are usually used to characterize the functional groups of carbon materials: Fourier infrared spectroscopy (FTIR), Raman spectroscopy (Raman), X-ray photoelectron spectroscopy (XPS), and nuclear magnetic resonance imaging technology (^13^C NMR). The carbon skeleton structures (carbon category), such as aliphatic carbon, olefinic carbon, aromatic carbon, and polyaromatic hydrocarbon, can be analyzed by FTIR, Raman, and ^13^C NMR. FTIR and Raman provide a fast measurement and an essential identification of the carbon’s surrounding environment. It should be noticed that Raman is more sensitive to carbon skeleton structure, while the aliphatic side chains and functional groups are usually characterized by FTIR and ^13^C NMR. ^13^C NMR could provide more structure parameters about the carbon skeleton and functional groups, but it is expensive. XPS can be used to analyze the relative content of functional groups, especially for oxygen-containing functional groups. It is unnecessary to use all these four methods together to determine the functional groups. One of these methods is adequate to obtain general information of the functional groups. Details of these four methods are discussed below.

##### Fourier Infrared Spectroscopy (FTIR)

FTIR has the advantages of simple operation and high resolution. It has been widely used in characterizing the evolution of functional groups of the coal and biomass char during high-temperature pyrolysis [56,57]. Coke is the residue product of coal after pyrolysis without air. Coke matrix is connected by amorphous carbon and ordered carbon structural units through bridge bonds. The ordered carbon structure, generally called aromatic core, is formed by condensation of the benzene ring, alicyclic ring, hydrogenated aromatic ring, and heterocyclic ring. The amorphous carbon and irregular structures are the alkyl side chain and an oxygen-containing functional group connecting to the aromatic nucleus [58]. 

**Figure 1:**
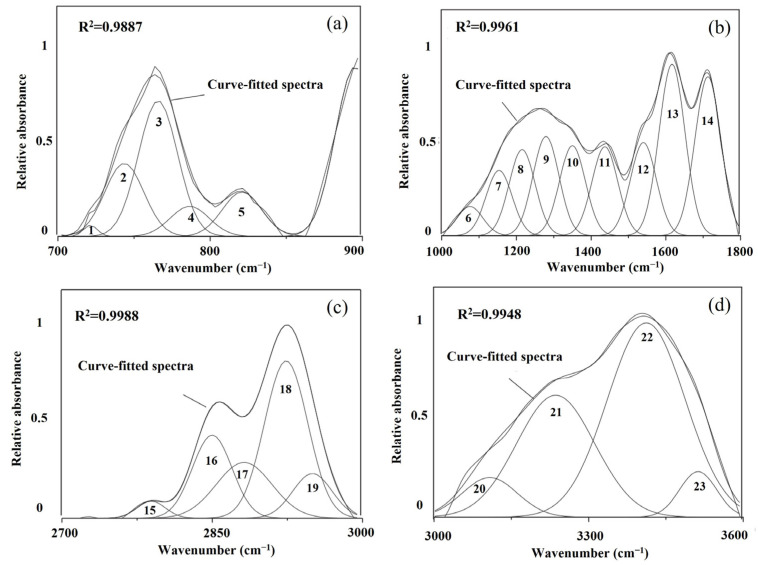
The FTIR spectrums of four sections: (**a**) aromatic structure; (**b**) oxygen-containing functional group; (**c**) aliphatic functional group; (**d**) hydroxyl group [60].

The key to the coke structure analysis by FTIR method lies in identifying absorption peak and its assignment. The different functional groups and relative contents in the coke structure can be determined by identifying the peak displacement. The typical FTIR spectrum can be divided into aromatic structure (700–900 cm^−1^), oxygen-containing functional group (1000–1800 cm^−1^), aliphatic functional group (2700–3000 cm^−1^), and hydroxyl group (3000–3600 cm^−1^) [59,60]. Feng et al. [60] illustrated the FTIR spectrum of these four structures in lignite, as shown in Figure 1. The peak-fitting information and parameters are listed in Table 1. The relative content of each functional group can be calculated by the ratio of the peak areas. The following ratios can be used as structure parameters to describe the functional groups semi-quantitatively [60]:

1. H_ar_/H (hydrogen aromaticity) is the ratio of H atoms in the aromatic compounds to the total amount of H atoms. This value is calculated as (areas of peak 1–5)/(areas of peak 1–5 + areas of peak 15–19);
2. CH_3_/CH_2_ represents the aliphatic chain length and degree of branching of the aliphatic side group (side chains attached to macromolecular structure). A lower value implies comparatively longer and straight chains. This value is calculated as (areas of peak 15, 17, and 19)/(areas of peak 16 and 18);
3. *f*_ar_ (aromatic–carbon ratio), representing the percentage of C atoms in sp^2^ hybrid relative to the total C. A higher value indicates higher aromaticity.

**Figure 2:**
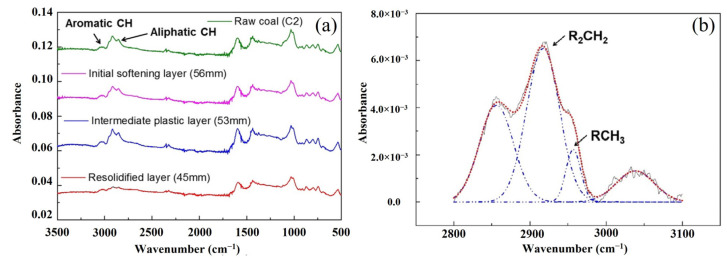
The FTIR spectrums of residue samples in the coke oven: (**a**) the residue sample in different positions of the coke oven; (**b**) an example of deconvolution of the spectra [57].

To investigate the changes in functional groups during coking, Lee et al. [57] used an in-situ sampling probe to obtain the residue sample in the different positions of the coke oven. The different positions of the coke oven simulated the different periods of the coking procedure. Figure 2a shows the FTIR spectrums of these samples, and Figure 2b shows an example of deconvolution of the spectra. The identification of absorption peak and its assignment agree with Table 1.

##### Raman Spectroscopy (Raman)

Raman is a kind of scattering spectrum. The scattering spectra with different frequencies of incident light can be analyzed to obtain the information of molecular vibration and rotation. The corresponding spectral data can be retrieved from the Raman spectrum database and applied to the study of molecular structure [61]. The Raman effect is independent of the laser wavelength, indicating that the laser wavelength range would have little influence on the Raman spectra. 

**Figure 3:**
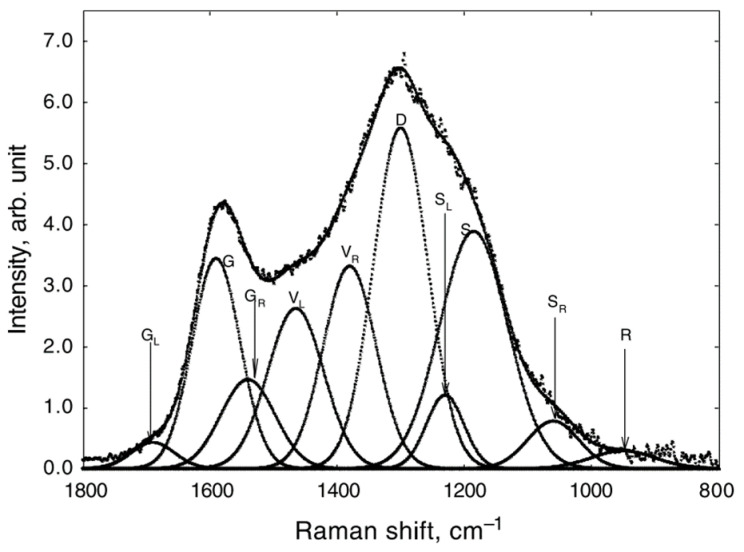
The fitted curves of the Roman spectrum of a carbonized sample [63].

The Raman spectra for carbon material mainly consist of G (graphite) and D (defect) bands, whose peaks locate around 1580–1600 cm^−1^ and 1350, respectively [62]. As for highly disordered carbonaceous material, such as coal char and coke, some structural information may be hidden in the overlapped area between these two bands. Li et al. [63] studied the structural evolution during the pyrolysis of brown coal by Raman spectroscopic method. To obtain detailed information about the skeletal carbon structures, the Raman spectra were curve-fitted in the range of 800 and 1800 cm^−1^. Figure 3 shows an example of a coal sample after carbonization at 700 °C. The ten typical bands are summarized in Table 2. 

**Figure 4:**
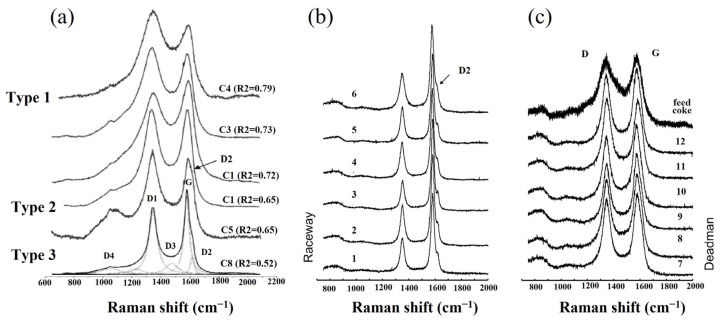
Raman spectra of cokes: (**a**) raw coke from industry [64]; (**b**,**c**) cokes from different parts of a blast furnace [65].

It is unnecessary to fit the Raman spectra with all the ten sub-bands. It depends on the shape of the raw curve. Rantitsch et al. [64] assessed the quality of the metallurgical cokes with varied CRI by Raman. Figure 4a shows the fitted Raman spectra of the cokes. To quantitively estimate the ordered carbon structure, the peak area ratio D1/(G + D1 + D2), namely R2, was defined. A smaller R2 value indicated a better microstructural order. It was found that the coke with a high reactivity contained a more disordered carbon structure. Rantitsch et al. [65] further analyzed the microstructural evolution of cokes from the deadman towards the raceway of a blast furnace. Figure 4b,c shows the Raman spectra of the cokes from different parts of a blast furnace. The coke from the raceway where the temperature was higher showed a well-ordered carbon structure.

##### X-ray Photoelectron Spectroscopy (XPS)

XPS is sensitive to the bonding states of C and O elements, such as graphitized C, C-O bond, C=O bond, and COO-bond. The fundamental use X-ray to transform the electrons within the atoms to free electrons. By measuring the generated energy of the photon, the binding energy of the electrons in the solid sample can be obtained. The binding energies of the elements in different chemical environments have slight differences, which are called chemical shifts. The state of the element can be determined by the magnitude of the chemical shift [66]. The summary of carbon functional groups and corresponding binding energy are listed in Table 3. 

**Figure 5:**
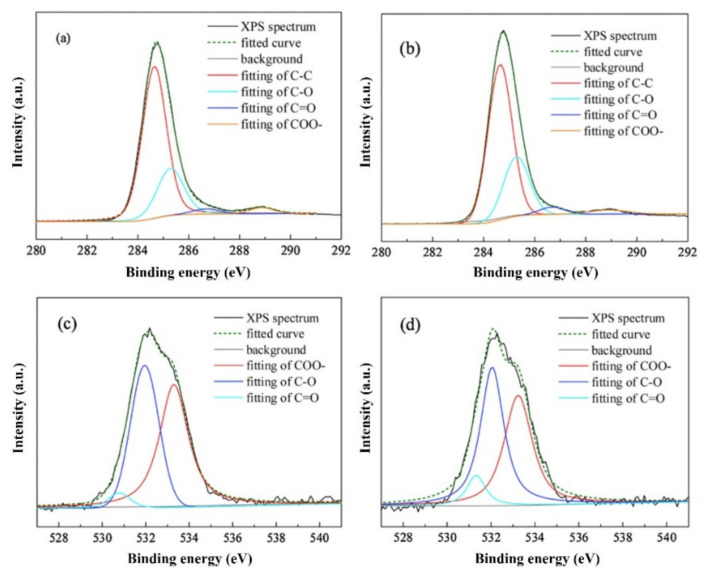
The XPS C1s and O1s spectra of cokes: (**a**) C1s of B0 coke; (**b**) C1s of B3 coke; (**c**) O1s of B0 coke; (**d**) O1s of B3 coke [67].

Liu et al. [67] investigated the influence of blend ratios on the coke morphology and composition. The atomic ratios of C and O in different functional group states were calculated by XPS-PEAK software. Figure 5 shows the typical XPS C1s and O1s spectra of the cokes. B0 and B3 are the cokes without and with 30% bio tar. The relative contents of different C and various classes of O in these two coke samples are presented in Table 4. It shows that most carbons are in the C-C and C-H forms. The main form of O in coke is phenol hydroxyl. 

##### Nuclear Magnetic Resonance Imaging Technology (^13^C NMR)

**Figure 6:**
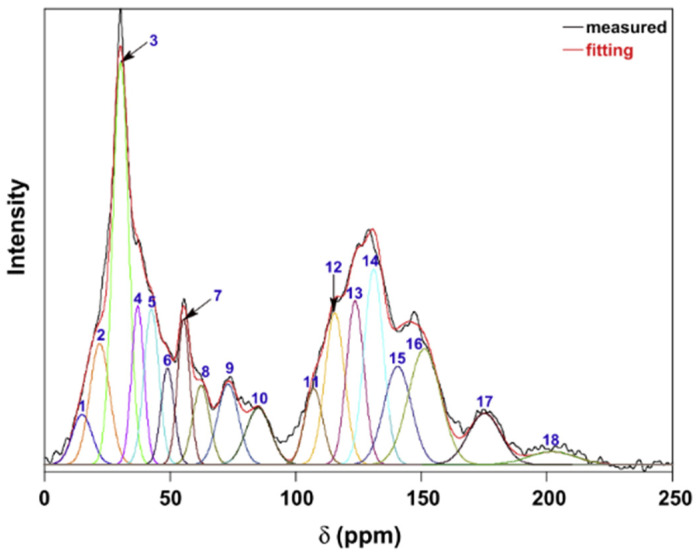
The ^13^C NMR spectra of lignite coal [68].

Few studies can be found discussing the ^13^C NMR spectra of coke. Most of the research focuses on the investigation of coal structure. ^13^C NMR is a direct and effective method to describe the carbon skeleton structure and functional groups of carbon material. The side chains and functional groups in the coke structure would decompose during the coking, where the assignments of different carbon types remain the same as coal structure. Li et al. [68] studied the carbon skeleton of coal and divided the spectra into three chemical regions: aliphatic carbon (0–90 ppm), aromatic carbons (90–170 ppm), and carbonyl carbons (170–220 ppm). The proportion of structural units in the overall structure can be obtained by the peak fit. Figure 6 shows a typical ^13^C NMR spectra of lignite coal. The chemical shift and molar content of different C types are summarized in Table 5. 

### 3.2. Physical Structure

Coke is a brittle material with a developed pore system composed of pore walls, pores, and cracks. During coking, the side chains on the aromatic core of coal continuously break off and decompose. Meanwhile, the aromatic core condenses and thickens to form a microcrystalline structure. Therefore, the basic unit of the coke pore wall is a microcrystallite structure [40]. The pore structure is usually characterized by image, X-ray CT imaging, mercury intrusion, and N_2_ gas adsorption methods. The microcrystallite structure is generally characterized by X-ray diffraction (XRD), high-resolution transmission electron microscopy (TEM/HRTEM), and Raman spectroscopy. 

#### 3.2.1. Pore Structure

**Figure 7:**
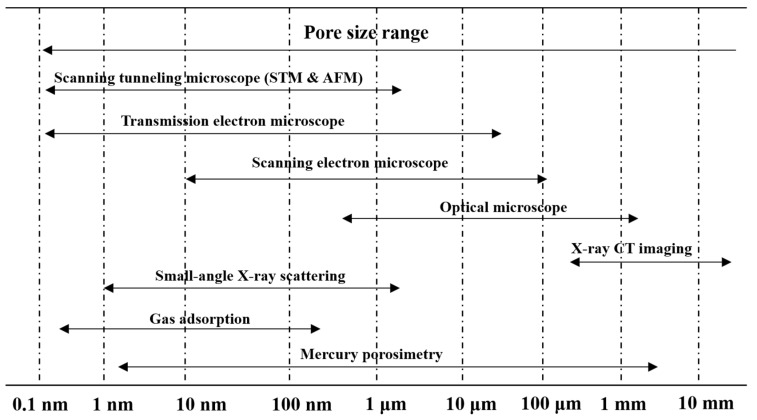
Identification techniques of pores in in solid materials.

Pores in solid materials have been identified by different techniques, mainly depending on their sizes. The methods for the identification of pores are summarized in Figure 7. The pores in the coke are generally in an extensive size range. Large pores on the surface of a coke can be observed using the microscope image method [69,70] and X-ray CT imaging technique [71,72,73]. X-ray CT imaging is a non-destructive detection method. However, due to the relatively low resolution, the microscope image method is still required for further analysis of pore structure. For micropores and mesopores, nitrogen adsorption and mercury intrusion methods are used to analyze the specific area and pore size distribution. The pore structures of coke on multiscale can be characterized comprehensively through the above methods.

##### Microscope Image Method

**Figure 8:**
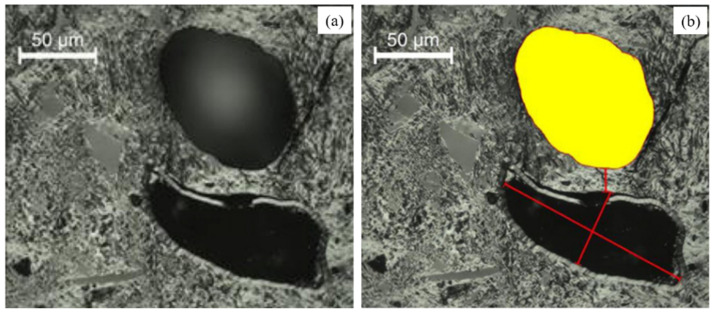
An example of the determination of pore parameters in a coke matrix: (**a**) original image; (**b**) measurement process [69].

Nyathi et al. [69] analyzed the pore structure in different sizes of coke by optical microscope. One hundred images were taken systematically for each sample. The image processing program can obtain the pore parameters, such as pore area, pore wall thickness, and pore diameter. Figure 8 shows an example of how to determine these pore parameters. The pore parameters of coke with different sizes are shown in Table 6. The pore wall thickness is the width of the carbon skeleton (<50 μm) between two pores. The particle size of the coke shows a minor influence on the determination of porosity via the microscope image method. Generally, the porosity and mean pore area increase with the coke size. At the same time, the mean thickness of the pore wall shows the opposite trend.

**Figure 9:**
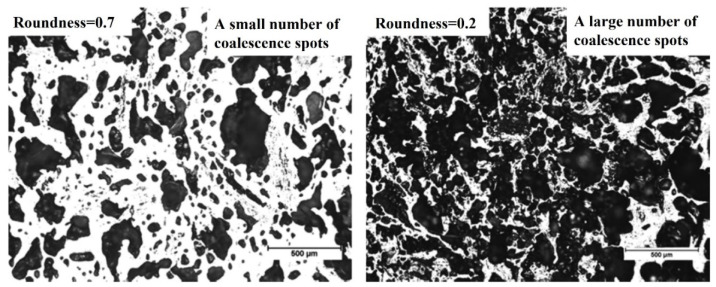
An example of the determination of pore parameters in a coke matrix [70].

Kubota et al. [74] and Xing et al. [70] defined a shape factor of the pore by roundness (R), as shown in Equation (1). The smaller the R-value, the more complicated the shape of pores. When R equals 1, it represents the pore shape is a circle. The pores with regular shape and larger roundness can resist more vital external breaks. It can be seen from Figure 9 that the pores with a roundness of 0.2 are more irregular in two-dimensional than those with a roundness of 0.7. The change of roundness is accompanied by the evolution of pore structures, such as the merge of pores and the collapse of the pore walls.

(1) $$R=\frac{4\pi S}{L^{2}}$$

where S and L are the areas and the circumference of a pore. Generally, coke with higher pore roundness and larger pore wall thickness shows higher cold drum strength and hot strength (CSR) [69,74,75].

**Figure 10:**
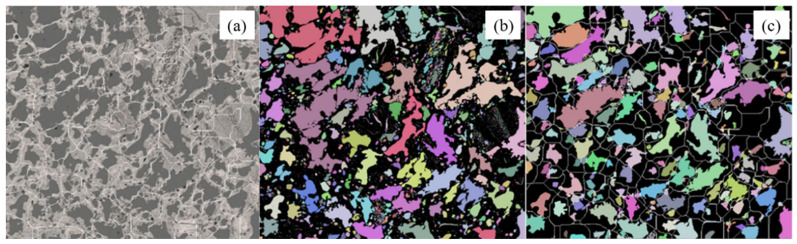
Pore automatic segmentation process: (**a**) the pore separation lines; (**b**) different pores with colors; (**c**) porosity color map after separation [78].

With the improvement of technology, the image method has been continuously enriched. To overcome the deviation of statistical results caused by manual intervention, many scholars [76,77] realized the automation image analysis of coke images. Figure 10 shows the automatic segmentation process of coke pores [78]. First, the separation lines were automatically generated based on pores. Then different pores were randomly colored, and some noise data were removed to form the color map. At last, it became easier to obtain the pore parameters of pores. 

##### X-ray CT Imaging

**Figure 11:**
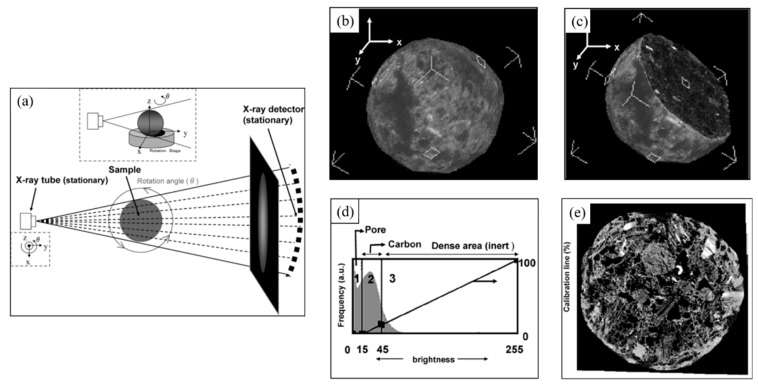
X-ray CT imaging process: (**a**) schematic diagram of X-ray CT; (**b**) 3D scanning of a spherical coke; (**c**) section diagram of a spherical coke; (**d**) picture brightness adjustment; (**e**) coke CT image [79]. Copy right CC BY-NC-ND 4.0.

Most of the coke samples need to be crushed, grounded, and polished for the image analysis method. The sample preparation process inevitably causes damage to the coke pore structure. Yuichiro et al. [79] used X-ray CT imaging technology, as shown in Figure 11a, to carry out non-destructive observation statistics on the 3D structure and porosity of coke. According to the density difference between coke matrix and pores, the coke 3D scanning diagram, as shown in Figure 11b–e, can be obtained, making it possible to observe the distribution and connection of coke pores in 3D space.

**Figure 12:**
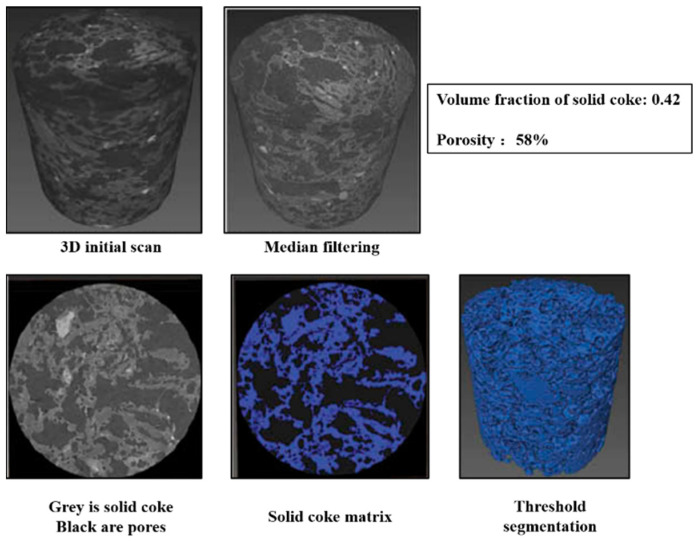
The pore distribution of coke samples in X-ray imaging [80].

Ghosh et al. [80] used X-ray CT imaging technology to visualize coke pore distribution in 3D space through threshold segmentation, as shown in Figure 12. The size information of coke pore structures can be calculated by the corresponding relationship between image pixels and actual size. X-ray CT imaging technology is based on the different densities of different materials in the tested sample and then shows different gray levels in the image; therefore, it is an effective method for analyzing coke pore structure. It should be pointed out that the optical texture of coke cannot be distinguished by this method [81]. 

##### Mercury Intrusion and Nitrogen Gas Adsorption Methods

PThe above two methods are limited to the analysis of macropores. For pores in nanoscale, mercury intrusion and nitrogen gas adsorption methods are good options. The mercury intrusion method has the advantage of measuring pores with a wide size range (5 nm–5 mm). However, due to the high toxicity of mercury, mercury intrusion is not widely used. The nitrogen gas adsorption method is used for determining the micropore distributions and specific surface area of coke. According to the nitrogen gas adsorption method, the sample is placed in liquid nitrogen after degassing treatment. The relation between pressures and the adsorption amount of nitrogen can be obtained. Through the nitrogen adsorption–desorption isotherms, the parameters of pores can be determined. Furthermore, the pore size distribution, the pore volume, and the surface area of the coke can be calculated.

#### 3.2.2. Microcrystallite Structure

The microcrystallite structure of coke is typically characterized by X-ray diffraction (XRD). The average size of coke crystallite can be calculated based on the XRD pattern. In addition, high-resolution transmission electron microscopy (TEM/HRTEM) can directly observe the lattice fringe of the coke matrix and determine the ordering degree of coke. Raman spectroscopy can provide content of graphite crystalline (ordered carbon structure) in coke.

##### X-ray Diffraction (XRD)

**Figure 13:**
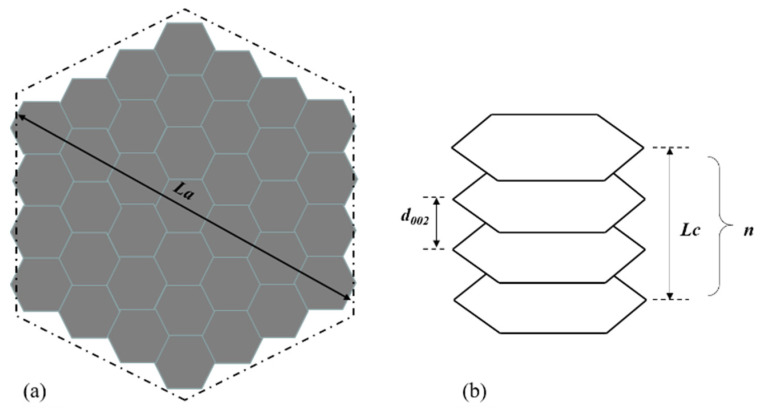
The geometry of an ideal microcrystallite: (**a**) top view; (**b**) side view.

**Figure 14:**
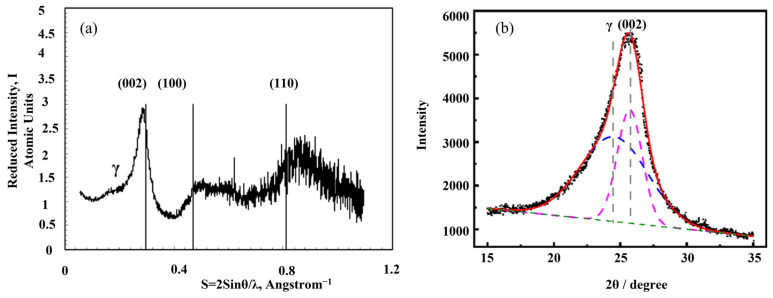
XRD patterns: (**a**) typical XRD pattern of carbon material [83]; (**b**) curve-fitting of XRD pattern of a coke [41].

XRD has been widely used to obtain the microcrystallite parameters of coke [38,82,83]. SiO_2_ and Al_2_O_3_ are the dominant inorganic minerals in the coke ash. If the contents of the inorganic minerals are not high, researchers usually ignore the relevant peaks in the XRD pattern. For a precise calculation of microcrystallite parameters, it is suggested to do demineralization treatment before a XRD test [84]. Li et al. [85] described the detailed procedure of the determination treatment by 6 vol% HCl and 40 wt% HF aqueous solution. As shown in Figure 13, several parameters have been defined to quantitively interpret the microcrystallite structure: the average stacking height (Lc) of crystalline, the average size (La) of crystalline layer, and the average spacing (d_002_) of crystalline [86]. The raw XRD pattern of carbon material, mainly coke, requires a data processing procedure to distinguish different interesting peaks. As shown in Figure 14a, the typical XRD pattern of carbon material contains four bands, namely (002), (100), (110), and γ. It should be noted that the x coordinate in Figure 14a is “2 sin θ/λ” instead of “2 θ”. Lu et al. [87] also used 2 sin θ/λ as the x coordinate in the XRD patterns. There is no strict rule about the x coordinate, which largely depends on the habit of the researchers. From Equation (3), the “2 sin θ/λ” represents the reciprocal of the average interlayer spacing at the specific Bragg angle. The (002) band, at around 24°–26°, represents the ordering degree of the aromatic planes in the microcrystallite. It is generally accepted that (002) band implies the average stack height of the aromatic planes of carbon microcrystallite. This characteristic band of graphite locates at 26°. If the (002) band is very sharp and close to 26°, it means that the carbon microcrystallite is highly ordered and shows similar microcrystallite as graphite. The (100) band at around 44° and the (110) band at about 81° both represent hexagonal ring structures. The higher and narrower these two peaks, the larger the hexagonal ring structure. The γ band, at around 16°–23°, is associated with aliphatic side chains or condensed saturated rings. Theoretically, the areas under the γ and (002) bands are believed to be equal to the number of aliphatic carbon atoms and aromatic carbon atoms, respectively [41]. Figure 14b shows a representative curve fitted with two Gaussian bands. All the data for the calculation of parameters can be obtained through the fitted curves.

According to the Scherrer equation [64,70], the average stacking height (*Lc*), the average size (*La*), the average interlayer spacing (*d_002_*), and the number of layers (*n*) of a crystalline can be calculated by Equations (2)–(5), as follows:

(2) $$Lc=\frac{0.89\lambda }{B_{002}\cos\theta_{002}}$$

(3) $$d_{002}=\frac{\lambda }{2\sin\theta_{002}}$$

(4) $$La=\frac{k_{1}\lambda }{B_{100}\cos\theta_{100}}$$

(5) $$n=\frac{Lc}{d_{002}}$$

where $B_{002}$ and $B_{100}$ are the full width at half maximum (FWHM) of (002) and (110) bands, respectively; $\theta_{002}$ and $\theta_{100}$ are the scanning angle of X-ray at (002) and (100) bands, respectively; λ is the wavelength of X-ray, normally λ = 1.5418 Å; $k_{1}$ is the correction coefficient, $k_{1}$ = 1.84. 

The graphitization degree, *r*_0_, of a coke can be calculated according to the *d*_002_, as shown in Equation (6) [88,89]. A higher value of r_0_ represents a higher ordering degree.

(6) $$r_{0}=\frac{\Delta d}{\Delta 0}=\frac{\left(3.440-d_{002}\right)\;Å}{\left(3.440-3.354\right)\;Å}$$

where 3.44 Å is the spacing of ideal none-graphitized carbon crystalline; $d_{002}$ is the average spacing of coke sample crystalline; 3.35400 Å is ideal graphitized carbon crystalline spacing.

##### Transmission Electron Microscopy (TEM)

**Figure 15:**
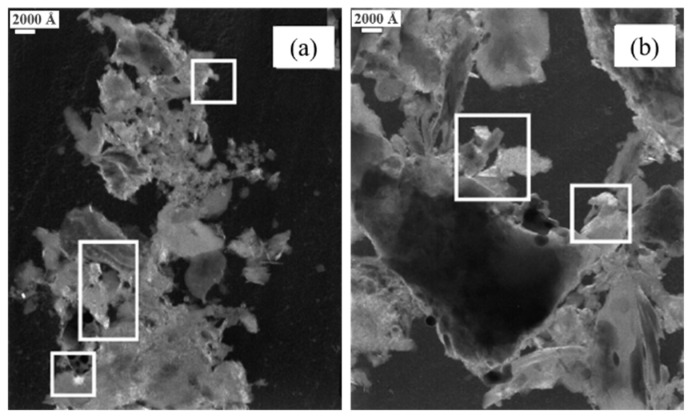
The MODs in the coke microregion: (**a**) without reaction; (**b**) reaction with CO_2_ [18].

TEM has a resolution of 0.1~0.2 nm and a magnification of 10^4^–10^6^ times, which can be used to observe the structure of the coke matrix microregion. Rouzaud et al. [90] found that the molecular oriented domain (MOD) in the coke was one key factor to the reactivity, especially at the beginning of the gasification when the porosity was closed. The MODs are graphite-like structures that mainly develop along the pore wall. TEM method requires the sample to be thin enough, which limits its application in a certain field. To solve the sample preparation problem, Pusz et al. [18] ground the coke sample in ethanol and then disperse the sample under ultrasonic bath treatment. The suspension liquid was placed on a copper grid covered by a carbon film. The sample can be used for TEM after the ethanol evaporates. As shown in Figure 15, the microstructural images of the coke can be obtained. It was found that after reaction with CO_2_, the molecular oriented domains (MODs) in the coke microregion became more extensive and more apparent, indicating that the microcrystallite is more orderly and tends to graphitization. The bright region in the white box is the MODs. 

##### High-Resolution Transmission Electron Microscopy (HRTEM)

HRTEM has a higher resolution than TEM and can observe internal structures, such as the lattice fringe, of coke. The lattice fringe groups that represent graphite layers can be extracted from the lattice image by image processing technology. The interplanar spacing can be clearly seen by light and dark stripes. According to the characteristic parameters of the fringe groups, including length, curvature, and inclination angle, the microcrystallite parameters, such as the size of microcrystallite and the spacing of planes, can be directly calculated. 

**Figure 16:**
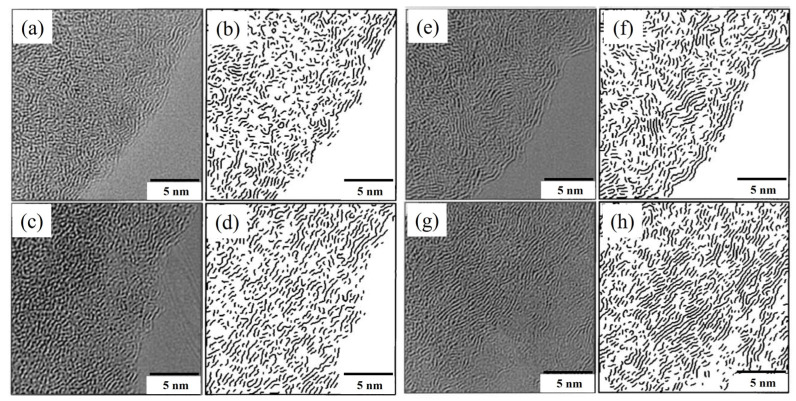
HRTEM image and stripe group: (**a**,**b**) original coal sample; (**c**,**d**) coal pyrolysis sample at 427 °C; (**e**,**f**) coal pyrolysis sample at 800 °C; (**g**,**h**) coal pyrolysis sample at 1200 °C [94].

Wu et al. [91,92] put forward a quantitative analysis method for the calculation of microcrystallite parameters of coke based on HRTEM images. The stripes in the HRTEM image represent the layers of microcrystallite. Each stripe in the image was identified and numbered. The stripes that met a particular condition were classified as a group of stripes. Each group was regarded as an oriented microcrystallite. Sharma et al. [93] tested the evolution of microcrystallite in phenol-formaldehyde resin char (PFC, a typical non-graphitized carbon) after pyrolysis at 1000 °C and 1400 °C. It was verified that the results from HRTEM were consistent with those obtained from XRD. The accuracy of HRTEM results was confirmed. Sharma et al. [94] further analyzed the evolution of microcrystallite in pulverized coal during coking at different temperatures. As shown in Figure 16, with the increase in pyrolysis temperature, the microcrystallite stripes tend to be distributed in parallel and arranged in an orderly manner. 

### 3.3. Optical Texture

The active components (vitrinite and liptinite) in coal would soften and melt during coking and form a continuous pore wall. The inert components (inertinite, opaque matter, and semi vitrinite) would remain in the pore walls together with the active softening components [95]. Under a polarized light microscope, the optical texture of coke is classified according to its microscopic characteristics (shape, size, and color). Coin et al. [96] classified the typical visual textures of coke as isotropic, mosaic, flake, fibrous, ribbon, anisotropic, and pyrolytic carbon. To simplify the characterization of optical texture, the optical textures of coke are only divided into isotropic texture and anisotropic texture. In that sense, all the optical textures except isotropic and inert can be considered anisotropic. Zhang et al. [45] evaluated the optical texture of six cokes from different plants according to Chinese standard YB/T 077-2017 (method for determination of coke visual structure). He found the coke with higher isotropic texture content exhibited higher reactivity. The optical texture contents and CRI of coke are listed in Table 7. 

The anisotropic degree (OTI) is another index to indicate the optical texture of coke, as expressed in Equation (7). In this case, further segmentation of the anisotropic texture type is needed.

(7) $$\mathrm{OTI}=\sum w_{i}{\left(AT\right)}_{i}$$

where $w_{i}$ is the content of each anisotropic texture type; ${\left(AT\right)}_{i}$ is the assignment value of each anisotropic texture type. The assignment value of each anisotropic type is listed in Table 8. A higher assignment value indicates a higher ordering structure. 

**Figure 17:**
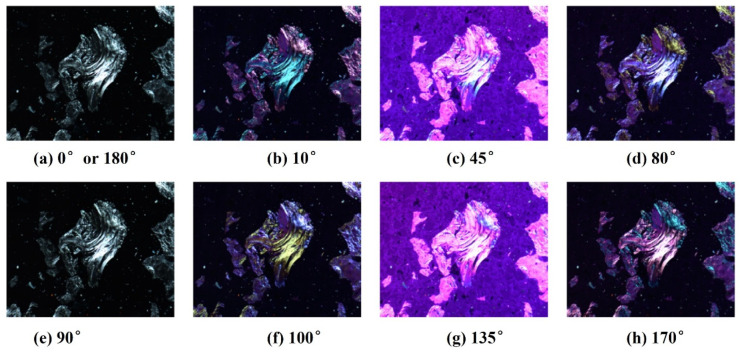
Colors of anisotropic texture in different analyzer angle positions: [97].

**Figure 18:**
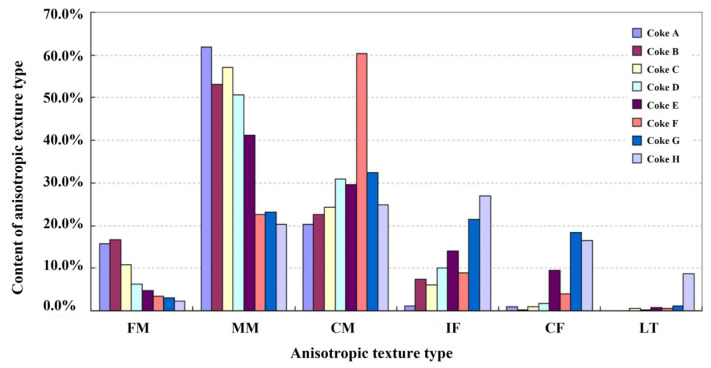
The contents of anisotropic texture types in eight cokes [97].

When the anisotropic texture is observed at different angles under the polarized light microscope, the interference colors will change; however, the interference color of the isotropic texture remains the same. This is the principle to distinguish these two kinds of optical textures. Sun et al. [97] developed an automatic measurement method for the visual texture of coke under the polarizing microscope. Figure 17 shows the optical images of the same field area of the coke from different analyzer angles. It should be noticed that Figure 17a,e present the images under orthogonal polarization. All the isotropic textures exhibit black due to the extinction light. The anisotropic texture exhibit brightness because the reflected light partly passes through the analyzer. In practice, only two different analyzer angles (10° and 170°) images are needed for further analysis. The further quantitative analysis was fulfilled by a computer program, conducting the automatic extraction, recognition, and classification of color-changed regions. Figure 18 shows the content of anisotropic texture type in cokes. Generally, the coke with a higher anisotropic degree offers lower CRI. 

## 4. Construction of Molecular Structure Model

It is essential to build a molecular structure model of coke to understand its physicochemical properties and reactivity. Due to the complexity, variety, and heterogeneity of structural features, conducting a universal molecular structure model for all kinds of coke is impossible. The structure of each type of coke is unique and can be constructed by a combination of structural characterization and computer programming.

**Figure 19:**
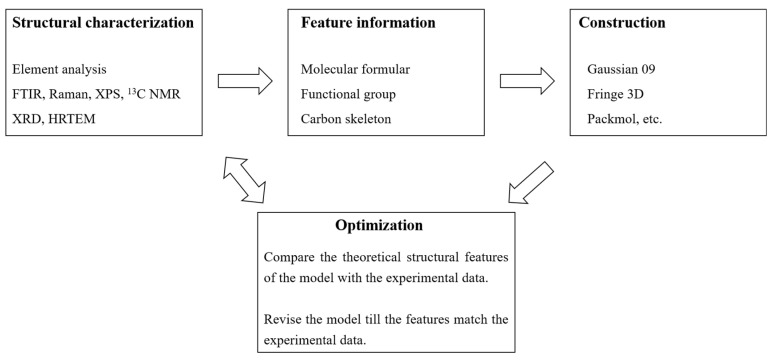
The general procedure of construction of a molecular structural model.

So far, there is plenty of literature about the molecular structure model of the coal [60,98,99]. However, limited information about the construction of the coke molecular model is available [55,100]. Both coal and coke are carbon materials with different carbon skeleton and functional groups. The general principle of the construction of the molecular structure model is similar and can be summarized, as shown in Figure 19. The key to the construction of the model is to obtain its basic structural units. After getting adequate structural parameters, the construction can be conducted by computer programs, such as Gaussian09 [101], Fringe 3D [102], and Packmol [103], etc. Afterward, the validation of the model must be confirmed. 

## 5. Conclusions

The relations between coke structures and properties are described in this review. The methods for a comprehensive characterization of the coke structures are summarized. 

The metallurgical properties of coke strongly depend on its microstructures. Generally, the coke matrix with a more disordered carbon structure shows low reactivity. The coke with higher pore roundness and larger pore wall thickness shows higher cold strength and hot strength. The reactivity of the coke decreases with the increase in Lc and La. The coke with a higher amount of anisotropic texture shows lower reactivity because of the higher ordering degree.

The current review divides the coke structures into chemical structure, physical structure, and optical texture, according to its relevant characteristics. The chemical structures of the coke can be characterized by ultimate analysis and FTIR, Raman, XPS, and ^13^C NMR, respectively. The pore structure is usually detected by the image method, the X-ray CT imaging technique, the mercury intrusion method, and the N_2_ gas adsorption method. The microcrystallite structure can be characterized by XRD, TEM/HRTEM, and Raman spectroscopy. The optical texture of the coke is classified according to its microscopic characteristics (shape, size, and color) under the polarized light microscope. The advantages and features of each method are introduced.

## 6. Outlook

The above results show that coke has many characterization methods, involving the structure characterization scale from macro to micro, from chemistry to physics. In China, we have already mastered most research methods for the characterization of coke structure, such as XRD, FTIR, Raman, XPS, the optical texture technique, the mercury intrusion method, and N_2_ gas adsorption method. However, some advanced and in-depth characterization methods are yet to be commonly used, such as TEM/HRTEM, 13C NMR, and the X-ray CT imaging technique, etc. Therefore, these advanced technologies need to be developed with emphasis. For a comprehensive understanding of coke structures, we recommend conducting chemical, physical, and optical texture scales characterization.

A molecular model of the coke can be constructed by a combination of structural characterization and computer programming. The molecular structural model helps to understand the mechanism of the diversities of coke performance and provides a possible approach to simulate the evolution of coke structure in extreme conditions. The evolution of coke structures and its properties can be explained on a molecular scale. Moreover, the properties of a coke can be predicted and designed based on the molecular model. However, very limited information about the construction of the coke molecular model is available. Therefore, more attention should be paid to the construction of the molecular structure model. In the near future, with the continuous improvement of technology, the performance of coke at various conditions can be simulated and predicted by computer methods, based on the molecular structure model.

## Figures and Tables

**Table 1:** Infrared bands belongings of the structures in lignite [60].

	Bands	Center/cm^−1^	Assignment
Aromatic structure	1	721	Mono-substituted benzene
	2	743	Di-substituted benzene
	3	766	Tri-substituted benzene
	4	787	Tri-substituted benzene
	5	819	Tetra-substituted benzene
Oxygen-containing and other carbon functional groups	6	1076	C-O-C alkyl ether
	7	1154	C-O aryl ether
	8	1215	C-O aryl ether
	9	1280	C-O phenols
	10	1350	CH3-Ar
	11	1437	CH3-, CH2-
	12	1539	Aromatic C=C
	13	1617	Aromatic C=C
	14	1713	CH3COOAr, C=O
Aliphatic functional groups	15	2789	O-CH_3_ or N-CH_3_
	16	2850	Sym.R_2_CH_2_
	17	2881	Sym.RCH_3_
	18	2924	Asym.R_2_CH_2_
	19	2950	Asym.CH_3_
Hydroxyl groups	20	3108	OH-N
	21	3236	Cyclic OH
	22	3411	OH-OH
	23	3512	OH-π

**Table 2:** Summary of the peak assignment [63].

Bands	Center/cm^−1^	Assignment
GL	1700	Carbonyl group C=O
G	1590	$\mathrm{Graphite}\;E_{2G}^{2}$; Aromatic ring quadrant breathing; alkene C=C
GR	1540	Aromatics with 3–5 rings; amorphous carbon structures
VL	1465	Methylene or methyl; semi-circle breathing of aromatic rings; amorphous carbon structures
VR	1380	Methyl group; semi-circle breathing of aromatic rings; amorphous carbon structures
D	1300	D band on highly ordered carbonaceous materials; C–C between aromatic rings and aromatics with not less than six rings
SL	1230	Aryl-alkyl ether; para-aromatics
S	1185	Caromatic-Calkyl; aromatic (aliphatic) ethers; C–C on hydroaromatic rings; hexagonal diamond carbon sp3; C–H on aromatic rings
SR	1060	C–H on aromatic rings; benzene (ortho-di-substituted) ring
R	960–800	C–C on alkanes and cyclic alkanes; C–H on aromatic rings

**Table 3:** Summary of carbon functional groups and corresponding binding energy.

Carbon Functional Group	C1s Binding Energy/eV	O1s Binding Energy/eV
C-C	284.4 ± 0.3	/
C-H	285.0 ± 0.3	/
C-O, C-O-C, C-OH	286.1 ± 0.2	532.8 ± 0.3
C=O	287.6 ± 0.3	531.3 ± 0.2
COO-	288.6 ± 0.4	534.1 ± 0.4

**Table 4:** C and O containing functional groups in B0 and B3 coke analyzed by XPS [67].

Element	Functional Group	Percentage/Atom %	
		B0	B3
C	C-C, C-H	70.50	63.17
	C-O, C-O-C, C-OH	24.05	28.44
	C=O	2.66	4.38
	COO-	2.79	4.01
O	C=O	10.09	11.55
	C-O, C-O-C, C-OH	60.61	56.07
	COO-	29.30	32.38

**Table 5:** Chemical shifts and molar contents of C types in lignite coal were analyzed by ^13^C NMR [68].

Band	Chemical Shift/ppm	C Type	Molar Content/%
Aliphatic			
1	15.0	Aliphatic CH3	2.4
2	22.0	Aliphatic CH3	5.9
3	30.3	Methylene	15.4
4	37.1	Methine	4.8
5	42.6	Quaternary	5.6
6–8	48–62	Oxy-methylene	10.8
9	70–90	Oxy-methine	7.5
10		Oxy-quaternary	
Aromatic			
11	107.1	*o*-Oxyaromatic protonated	3.3
12	115.5	*o*-Oxyaromatic branched	7.2
13	123.7	Aromatic protonated	6.9
14	131.0	Aromatic branched	9.1
15	140.6	Aromatic bridgehead	6.5
16	151.2	Oxygen-substituted aromatic	9.1
Carbonyl			
17	175.1	Carbonyl in carboxyl and ester	4.0
18	202.1	Carbonyl in ketone and aldehyde	1.5

**Table 6:** Pore parameters of coke with different sizes by optical microscope analysis [69].

Particle Size Range/mm	Porosity/%	Mean Pore Area/μm^2^	Mean Thickness of Pore Wall ^a^/μm
0.75–0.85	42	573	17.25
1.0–1.18	45	650	16.85
2.8–4.7	47	772	12.85
15–16	49	775	12.11

^a^ Mean thickness of the pore wall is measured manually.

**Table 7:** The optical texture contents and CRI value [45].

Coke	*∑ISO* ^a^/%	CRI/%	Coke	*∑ISO*/%	CRI/%
C1	28.8	18.4	C4	47.4	34.4
C2	31.0	21.3	C5	45.3	36.5
C3	23.0	20.6	C6	79.9	46.2

^a^*∑ISO* = % Isotropic + %Inert.

**Table 8:** Assignment value of each anisotropic type [97].

Anisotropic Texture Type	Assignment	Anisotropic Texture Type	Assignment
Fine mosaic (FM)	1	Incompletely fibrous (IF)	4
Medium mosaic (MM)	2	Completely fibrous (CF)	5
Coarse mosaic (CM)	3	Leaflet texture (LT)	6

## Data Availability

Data sharing not applicable. No new data were created or analyzed in this study. Data sharing is not applicable to this article.
